# Poster Session I - A125THE ROLE OF IMMIGRATION STATUS IN GASTROENTEROLOGY REFERRALS

**DOI:** 10.1093/jcag/gwaf042.125

**Published:** 2026-02-13

**Authors:** H R Tran, A Sajadi Naeini, Y Xu, S Bharatselvam, M Cino, C Parker, T Jeyalingam, Y Tse, J P Allard, P D James, P Rossos, F Habal, A Kohansal, L Liu, A N Sasson, P Tandon

**Affiliations:** Medicine, McMaster University Faculty of Health Sciences, Hamilton, ON, Canada; Medicine, McMaster University Faculty of Health Sciences, Hamilton, ON, Canada; University of Toronto, Toronto, ON, Canada; University of Toronto, Toronto, ON, Canada; University of Toronto, Toronto, ON, Canada; University of Toronto, Toronto, ON, Canada; University of Toronto, Toronto, ON, Canada; University of Toronto, Toronto, ON, Canada; University of Toronto, Toronto, ON, Canada; University of Toronto, Toronto, ON, Canada; University of Toronto, Toronto, ON, Canada; University of Toronto, Toronto, ON, Canada; University of Toronto, Toronto, ON, Canada; University of Toronto, Toronto, ON, Canada; University of Toronto, Toronto, ON, Canada; University of Toronto, Toronto, ON, Canada

## Abstract

**Background:**

Immigration status is a social determinant of health that affects access to health services. Gastroenterology (GI) wait times in Ontario are amongst the longest for medical subspecialties. The effect of immigration status on wait times for GI assessment and mental health have yet to be investigated for non-inflammatory bowel disease (IBD) gastrointestinal diseases.

**Aims:**

To determine the association between immigration status and wait time for GI consultation at a tertiary care referral center.

**Methods:**

Retrospective chart review of referral data was conducted on adults age ≥18 followed at two GI clinics in Toronto, Ontario with available immigration status up to June 11, 2025. Referral and consult dates, referring provider specialty, and reason for referral data were collected. Immigration status; sex; race; rurality of residence; and scores from standardized anxiety, depression, and stress questionnaires were extracted from a previously collected data registry. The primary endpoint was wait time in weeks from date of referral to date of first consult visit at the GI clinic. Mann-Whitney U-Test was used to compare median wait times and mental health questionnaire scores by immigration status.

**Results:**

145 patients were included in this interim analysis. 95 (66%) were Canadian-born (CB) and 50 (33%) reported prior immigration. CB and immigrant (I) cohorts both mostly comprised of females (p > 0.99), living in urban residences (p = 0.58), and of White European ethnicity (CB: n = 80, 84%; I: n = 23, 46%; p < 0.001). Overall median wait time was 9.4 weeks (interquartile range (IQR) 4.9-19.6). Family Medicine was the most common referring provider specialty among I patients (64%), whereas referrals from Gastroenterology were the most common among CB patients (39%). Among both CB and I cohorts, the most common reasons for referral were miscellaneous (CB: 47%; I: 42%). Screening colonoscopy (CB: 6.3%; I: 10%), active IBD (CB: 15%; I: 10%), and chronic constipation/diarrhea (CB: 6.3%; I: 8%) were other common reasons for referral in both cohorts. In comparisons of CB and I patients, there was no difference in median wait time (CB: 10.6 weeks (IQR 4.7-20.9); I: 8.4 weeks (IQR 5.4-14.3); p = 0.36). There was no difference in median scores for PHQ-9 for depression (p = 0.93), GAD7 for anxiety (p = 0.94), and PSS for perceived stress (p = 0.54).

**Conclusions:**

In this interim analysis, there appeared to be no significant difference in wait times for GI assessment and mental health scores between immigrants and non-immigrants. Future analyses will examine the association of wait time with reason for referral, concurrent diagnosis of IBD, duration of residence in Canada, mental health scores, and workplace activity limitation.

**Funding Agencies:**

None

